# Supplementary material for: Neural Correlates of Mobility in Children with Cerebral Palsy: A Systematic Review
Source: Int J Environ Res Public Health. 2024 Aug 7;21(8):1039. doi: 10.3390/ijerph21081039 (PMC11354175; doi:10.3390/ijerph21081039)
Supplement: Supplementary file 1 [file ijerph-21-01039-s001.zip › ijerph-3078367-supplementary.pdf]

Supplementary Material

Search strategy:

PUBMED

(((("cerebral palsy"[MeSH Terms] OR ("cerebral"[All Fields] AND "palsy"[All Fields]) OR "cerebral palsy"[All Fields] OR (("brain"[MeSH Terms] OR "brain"[All Fields] OR "brains"[All Fields] OR "brain s"[All Fields]) AND ("paralysis"[MeSH Terms] OR "paralysis"[All Fields] OR "palsied"[All Fields] OR "palsies"[All Fields] OR "palsy"[All Fields])) OR (("brain"[MeSH Terms] OR "brain"[All Fields] OR "brains"[All Fields] OR "brain s"[All Fields]) AND ("paralysing"[All Fields] OR "paralysis"[MeSH Terms] OR "paralysis"[All Fields] OR "paralyse"[All Fields] OR "paralysed"[All Fields] OR "paralyses"[All Fields])) OR (("central"[All Fields] OR "centrally"[All Fields] OR "centrals"[All Fields]) AND ("paralysis"[MeSH Terms] OR "paralysis"[All Fields] OR "palsied"[All Fields] OR "palsies"[All Fields] OR "palsy"[All Fields])) OR (("central"[All Fields] OR "centrally"[All Fields] OR "centrals"[All Fields]) AND ("paralysing"[All Fields] OR "paralysis"[MeSH Terms] OR "paralysis"[All Fields] OR "paralyse"[All Fields] OR "paralysed"[All Fields] OR "paralyses"[All Fields])) OR ("cerebral palsy"[MeSH Terms] OR ("cerebral"[All Fields] AND "palsy"[All Fields]) OR "cerebral palsy"[All Fields]) OR ("cerebral palsy"[MeSH Terms] OR ("cerebral"[All Fields] AND "palsy"[All Fields]) OR "cerebral palsy"[All Fields] OR ("cerebral"[All Fields] AND "paralysis"[All Fields]) OR "cerebral paralysis"[All Fields]) OR (("cerebrally"[All Fields] OR "cerebrum"[MeSH Terms] OR "cerebrum"[All Fields] OR "cerebral"[All Fields] OR "brain"[MeSH Terms] OR "brain"[All Fields]) AND ("paresis"[MeSH Terms] OR "paresis"[All Fields] OR "pareses"[All Fields])) OR (("diplegia"[All Fields] OR "diplegias"[All Fields]) AND "spastica"[All Fields]) OR ("encephalopathia"[All Fields] AND "infantilis"[All Fields]) OR ("cerebral palsy"[MeSH Terms] OR ("cerebral"[All Fields] AND "palsy"[All Fields]) OR "cerebral palsy"[All Fields] OR ("palsy"[All Fields] AND "cerebral"[All Fields]) OR "palsy cerebral"[All Fields]) OR ("cerebral palsy"[MeSH Terms] OR ("cerebral"[All Fields] AND "palsy"[All Fields]) OR "cerebral palsy"[All Fields] OR ("spastic"[All Fields] AND "diplegia"[All Fields]) OR "spastic diplegia"[All Fields])) AND ("child"[MeSH Terms] OR "child"[All Fields] OR "children"[All Fields] OR "child s"[All Fields] OR "children s"[All Fields] OR "childrens"[All Fields] OR "childs"[All Fields] OR ("child"[MeSH Terms] OR "child"[All Fields] OR "children"[All Fields] OR "child s"[All Fields] OR "children s"[All Fields] OR "childrens"[All Fields] OR "childs"[All Fields]) OR ("toddler"[All Fields] OR "toddler s"[All Fields] OR "toddlers"[All Fields]) OR ("toddler"[All Fields] OR "toddler s"[All Fields] OR "toddlers"[All Fields]) OR ("adolescences"[All Fields] OR "adolescency"[All Fields] OR "adolescent"[MeSH Terms] OR "adolescent"[All Fields] OR "adolescence"[All Fields] OR "adolescents"[All Fields] OR "adolescent s"[All Fields]) OR ("adolescent"[MeSH Terms] OR "adolescent"[All Fields] OR "teenage"[All Fields] OR "teenager"[All Fields] OR "teenagers"[All Fields] OR "teenaged"[All Fields] OR "teenager s"[All Fields] OR "teenages"[All Fields]) OR ("adolescences"[All Fields] OR "adolescency"[All Fields] OR "adolescent"[MeSH Terms] OR "adolescent"[All Fields] OR "adolescence"[All Fields] OR "adolescents"[All Fields] OR "adolescent s"[All Fields]) OR ("infant"[MeSH Terms] OR "infant"[All Fields] OR "infants"[All Fields] OR "infant s"[All Fields]))) NOT ("adult"[MeSH Terms] OR "adult"[All Fields]

OR "adults"[All Fields] OR "adult s"[All Fields] OR ("adult"[MeSH Terms] OR "adult"[All Fields] OR "adults"[All Fields] OR "adult s"[All Fields]) OR "grown ups"[All Fields] OR "grownup"[All Fields] OR "grownups"[All Fields] OR ("aged"[MeSH Terms] OR "aged"[All Fields]) OR (("aged"[MeSH Terms] OR "aged"[All Fields]) AND ("patient s"[All Fields] OR "patients"[MeSH Terms] OR "patients"[All Fields] OR "patient"[All Fields] OR "patients s"[All Fields])) OR (("aged"[MeSH Terms] OR "aged"[All Fields]) AND ("people s"[All Fields] OR "peopled"[All Fields] OR "peopling"[All Fields] OR "persons"[MeSH Terms] OR "persons"[All Fields] OR "people"[All Fields] OR "peoples"[All Fields])) OR ("aged"[MeSH Terms] OR "aged"[All Fields] OR ("aged"[All Fields] AND "person"[All Fields]) OR "aged person"[All Fields]) OR (("aged"[MeSH Terms] OR "aged"[All Fields]) AND ("subject"[All Fields] OR "subject s"[All Fields] OR "subjects"[All Fields] OR "subjects s"[All Fields])) OR ("aged"[MeSH Terms] OR "aged"[All Fields] OR "elderly"[All Fields] OR "elderlies"[All Fields] OR "elderly s"[All Fields] OR "elderlys"[All Fields]) OR (("aged"[MeSH Terms] OR "aged"[All Fields] OR "elderly"[All Fields] OR "elderlies"[All Fields] OR "elderly s"[All Fields] OR "elderlys"[All Fields]) AND ("patient s"[All Fields] OR "patients"[MeSH Terms] OR "patients"[All Fields] OR "patient"[All Fields] OR "patients s"[All Fields])) OR (("aged"[MeSH Terms] OR "aged"[All Fields] OR "elderly"[All Fields] OR "elderlies"[All Fields] OR "elderly s"[All Fields] OR "elderlys"[All Fields]) AND ("people s"[All Fields] OR "peopled"[All Fields] OR "peopling"[All Fields] OR "persons"[MeSH Terms] OR "persons"[All Fields] OR "people"[All Fields] OR "peoples"[All Fields])) OR ("aged"[MeSH Terms] OR "aged"[All Fields] OR ("elderly"[All Fields] AND "person"[All Fields]) OR "elderly person"[All Fields]) OR (("aged"[MeSH Terms] OR "aged"[All Fields] OR "elderly"[All Fields] OR "elderlies"[All Fields] OR "elderly s"[All Fields] OR "elderlys"[All Fields]) AND ("subject"[All Fields] OR "subject s"[All Fields] OR "subjects"[All Fields] OR "subjects s"[All Fields])) OR ("aged"[MeSH Terms] OR "aged"[All Fields] OR ("senior"[All Fields] AND "citizen"[All Fields]) OR "senior citizen"[All Fields]) OR "senium"[All Fields] OR ("aged"[MeSH Terms] OR "aged"[All Fields] OR ("older"[All Fields] AND "adults"[All Fields]) OR "older adults"[All Fields]) OR (("older"[All Fields] OR "olders"[All Fields]) AND ("people s"[All Fields] OR "peopled"[All Fields] OR "peopling"[All Fields] OR "persons"[MeSH Terms] OR "persons"[All Fields] OR "people"[All Fields] OR "peoples"[All Fields])) OR ("infant, newborn"[MeSH Terms] OR ("infant"[All Fields] AND "newborn"[All Fields]) OR "newborn infant"[All Fields] OR "newborn"[All Fields] OR "newborns"[All Fields] OR "newborn s"[All Fields]) OR ("animals, newborn"[MeSH Terms] OR ("animals"[All Fields] AND "newborn"[All Fields]) OR "newborn animals"[All Fields] OR ("animals"[All Fields] AND "newborn"[All Fields]) OR "animals newborn"[All Fields]) OR (("human s"[All Fields] OR "humans"[MeSH Terms] OR "humans"[All Fields] OR "human"[All Fields]) AND ("infant, newborn"[MeSH Terms] OR ("infant"[All Fields] AND "newborn"[All Fields]) OR "newborn infant"[All Fields] OR "neonatal"[All Fields] OR "neonate"[All Fields] OR "neonates"[All Fields] OR "neonatality"[All Fields] OR "neonatals"[All Fields] OR "neonate s"[All Fields])) OR (("human s"[All Fields] OR "humans"[MeSH Terms] OR "humans"[All Fields] OR "human"[All Fields]) AND ("infant, newborn"[MeSH Terms] OR ("infant"[All Fields] AND "newborn"[All Fields]) OR "newborn infant"[All Fields] OR "newborn"[All Fields] OR "newborns"[All Fields] OR "newborn s"[All Fields])) OR ("animals, newborn"[MeSH Terms] OR ("animals"[All Fields] AND "newborn"[All

Fields]) OR "newborn animals"[All Fields] OR ("neonatal"[All Fields] AND "animal"[All Fields]) OR "neonatal animal"[All Fields]) OR ("infant, newborn"[MeSH Terms] OR ("infant"[All Fields] AND "newborn"[All Fields]) OR "newborn infant"[All Fields] OR "neonatal"[All Fields] OR "neonate"[All Fields] OR "neonates"[All Fields] OR "neonatality"[All Fields] OR "neonatals"[All Fields] OR "neonate s"[All Fields]) OR (("infant, newborn"[MeSH Terms] OR ("infant"[All Fields] AND "newborn"[All Fields]) OR "newborn infant"[All Fields] OR "neonatal"[All Fields] OR "neonate"[All Fields] OR "neonates"[All Fields] OR "neonatality"[All Fields] OR "neonatals"[All Fields] OR "neonate s"[All Fields]) AND ("animals"[MeSH Terms:noexp] OR "animal"[All Fields])) OR "neonatus"[All Fields] OR ("infant, newborn"[MeSH Terms] OR ("infant"[All Fields] AND "newborn"[All Fields]) OR "newborn infant"[All Fields] OR "newborn"[All Fields] OR "newborns"[All Fields] OR "newborn s"[All Fields]) OR ("animals, newborn"[MeSH Terms] OR ("animals"[All Fields] AND "newborn"[All Fields]) OR "newborn animals"[All Fields] OR ("newborn"[All Fields] AND "animal"[All Fields]) OR "newborn animal"[All Fields]) OR ("animals, newborn"[MeSH Terms] OR ("animals"[All Fields] AND "newborn"[All Fields]) OR "newborn animals"[All Fields] OR ("newborn"[All Fields] AND "animals"[All Fields])) OR ("infant, newborn"[MeSH Terms] OR ("infant"[All Fields] AND "newborn"[All Fields]) OR "newborn infant"[All Fields] OR ("newborn"[All Fields] AND "baby"[All Fields]) OR "newborn baby"[All Fields]) OR (("infant, newborn"[MeSH Terms] OR ("infant"[All Fields] AND "newborn"[All Fields]) OR "newborn infant"[All Fields] OR "newborn"[All Fields] OR "newborns"[All Fields] OR "newborn s"[All Fields]) AND ("child"[MeSH Terms] OR "child"[All Fields] OR "children"[All Fields] OR "child s"[All Fields] OR "children s"[All Fields] OR "childrens"[All Fields] OR "childs"[All Fields])) OR ("infant, newborn"[MeSH Terms] OR ("infant"[All Fields] AND "newborn"[All Fields]) OR "newborn infant"[All Fields] OR ("newborn"[All Fields] AND "infant"[All Fields])) OR ("newly"[All Fields] AND ("parturition"[MeSH Terms] OR "parturition"[All Fields] OR "born"[All Fields]) AND ("animals"[MeSH Terms:noexp] OR "animal"[All Fields])) OR ("newly"[All Fields] AND ("parturition"[MeSH Terms] OR "parturition"[All Fields] OR "born"[All Fields]) AND ("infant, newborn"[MeSH Terms] OR ("infant"[All Fields] AND "newborn"[All Fields]) OR "newborn infant"[All Fields] OR "baby"[All Fields] OR "infant"[MeSH Terms] OR "infant"[All Fields])) OR ("newly"[All Fields] AND ("parturition"[MeSH Terms] OR "parturition"[All Fields] OR "born"[All Fields]) AND ("child"[MeSH Terms] OR "child"[All Fields] OR "children"[All Fields] OR "child s"[All Fields] OR "children s"[All Fields] OR "childrens"[All Fields] OR "childs"[All Fields])) OR ("newly"[All Fields] AND ("parturition"[MeSH Terms] OR "parturition"[All Fields] OR "born"[All Fields]) AND ("infant"[MeSH Terms] OR "infant"[All Fields] OR "infants"[All Fields] OR "infant s"[All Fields])) OR ("fetus"[MeSH Terms] OR "fetus"[All Fields] OR "fetuses"[All Fields] OR "fetus s"[All Fields] OR "foetu"[All Fields] OR "foetus"[All Fields]) OR (("fetale"[All Fields] OR "fetally"[All Fields] OR "fetals"[All Fields] OR "fetus"[MeSH Terms] OR "fetus"[All Fields] OR "fetal"[All Fields] OR "foetal"[All Fields]) AND ("period"[All Fields] OR "periodic"[All Fields] OR "periodical"[All Fields] OR "periodically"[All Fields] OR "periodicals"[All Fields] OR "periodicity"[MeSH Terms] OR "periodicity"[All Fields] OR "periodicities"[All Fields] OR "periods"[All Fields])) OR (("fetale"[All Fields] OR "fetally"[All Fields] OR "fetals"[All Fields] OR "fetus"[MeSH Terms] OR "fetus"[All Fields] OR "fetal"[All Fields] OR "foetal"[All Fields]) AND ("phase"[All Fields] OR

"phase s"[All Fields] OR "phases"[All Fields])) OR (("fetale"[All Fields] OR "fetally"[All Fields] OR "fetals"[All Fields] OR "fetus"[MeSH Terms] OR "fetus"[All Fields] OR "fetal"[All Fields] OR "foetal"[All Fields]) AND ("stage"[All Fields] OR "staged"[All Fields] OR "stages"[All Fields] OR "staging"[All Fields] OR "stagings"[All Fields])) OR ("fetus"[MeSH Terms] OR "fetus"[All Fields] OR "fetuses"[All Fields] OR "fetus s"[All Fields] OR "foetu"[All Fields] OR "foetus"[All Fields]) OR (("fetale"[All Fields] OR "fetally"[All Fields] OR "fetals"[All Fields] OR "fetus"[MeSH Terms] OR "fetus"[All Fields] OR "fetal"[All Fields] OR "foetal"[All Fields]) AND ("period"[All Fields] OR "periodic"[All Fields] OR "periodical"[All Fields] OR "periodically"[All Fields] OR "periodicals"[All Fields] OR "periodicity"[MeSH Terms] OR "periodicity"[All Fields] OR "periodicities"[All Fields] OR "periods"[All Fields])) OR (("fetale"[All Fields] OR "fetally"[All Fields] OR "fetals"[All Fields] OR "fetus"[MeSH Terms] OR "fetus"[All Fields] OR "fetal"[All Fields] OR "foetal"[All Fields]) AND ("phase"[All Fields] OR "phase s"[All Fields] OR "phases"[All Fields])) OR (("fetale"[All Fields] OR "fetally"[All Fields] OR "fetals"[All Fields] OR "fetus"[MeSH Terms] OR "fetus"[All Fields] OR "fetal"[All Fields] OR "foetal"[All Fields]) AND ("stage"[All Fields] OR "staged"[All Fields] OR "stages"[All Fields] OR "staging"[All Fields] OR "stagings"[All Fields])) OR ("fetus"[MeSH Terms] OR "fetus"[All Fields] OR "fetuses"[All Fields] OR "fetus s"[All Fields] OR "foetu"[All Fields] OR "foetus"[All Fields]))) AND ((("biped"[All Fields] OR "bipeds"[All Fields]) AND ("gait"[MeSH Terms] OR "gait"[All Fields])) OR ("gait"[MeSH Terms] OR "gait"[All Fields]) OR ("gait analysis"[MeSH Terms] OR ("gait"[All Fields] AND "analysis"[All Fields]) OR "gait analysis"[All Fields]) OR (("gait"[MeSH Terms] OR "gait"[All Fields]) AND ("education"[MeSH Subheading] OR "education"[All Fields] OR "training"[All Fields] OR "education"[MeSH Terms] OR "train"[All Fields] OR "train s"[All Fields] OR "trained"[All Fields] OR "training s"[All Fields] OR "trainings"[All Fields] OR "trains"[All Fields])) OR (("behavior"[MeSH Terms] OR "behavior"[All Fields] OR "behavioral"[All Fields] OR "behavioural"[All Fields] OR "behavior s"[All Fields] OR "behaviorally"[All Fields] OR "behaviour"[All Fields] OR "behaviourally"[All Fields] OR "behaviours"[All Fields] OR "behaviors"[All Fields] OR "pattern"[All Fields] OR "pattern s"[All Fields] OR "patternability"[All Fields] OR "patternable"[All Fields] OR "patterned"[All Fields] OR "patterning"[All Fields] OR "patternings"[All Fields] OR "patterns"[All Fields]) AND ("walked"[All Fields] OR "walking"[MeSH Terms] OR "walking"[All Fields] OR "walks"[All Fields])) OR (("walked"[All Fields] OR "walking"[MeSH Terms] OR "walking"[All Fields] OR "walks"[All Fields]) AND ("behavior"[MeSH Terms] OR "behavior"[All Fields] OR "behavioral"[All Fields] OR "behavioural"[All Fields] OR "behavior s"[All Fields] OR "behaviorally"[All Fields] OR "behaviour"[All Fields] OR "behaviourally"[All Fields] OR "behaviours"[All Fields] OR "behaviors"[All Fields] OR "pattern"[All Fields] OR "pattern s"[All Fields] OR "patternability"[All Fields] OR "patternable"[All Fields] OR "patterned"[All Fields] OR "patterning"[All Fields] OR "patternings"[All Fields] OR "patterns"[All Fields])) OR ("locomote"[All Fields] OR "locomoted"[All Fields] OR "locomoter"[All Fields] OR "locomotes"[All Fields] OR "locomoting"[All Fields] OR "locomotion"[MeSH Terms] OR "locomotion"[All Fields] OR "locomotions"[All Fields] OR "locomotive"[All Fields] OR "locomotives"[All Fields]) OR (("behavior"[MeSH Terms] OR "behavior"[All Fields] OR "behavioral"[All Fields] OR "behavioural"[All Fields] OR "behavior s"[All Fields] OR "behaviorally"[All Fields] OR "behaviour"[All Fields] OR

"behaviourally"[All Fields] OR "behaviours"[All Fields] OR "behaviors"[All Fields] OR "pattern"[All Fields] OR "pattern s"[All Fields] OR "patternability"[All Fields] OR "patternable"[All Fields] OR "patterned"[All Fields] OR "patterning"[All Fields] OR "patternings"[All Fields] OR "patterns"[All Fields]) AND ("locomotor"[All Fields] OR "locomotors"[All Fields])) OR (("behavior"[MeSH Terms] OR "behavior"[All Fields] OR "behavioral"[All Fields] OR "behavioural"[All Fields] OR "behavior s"[All Fields] OR "behaviorally"[All Fields] OR "behaviour"[All Fields] OR "behaviourally"[All Fields] OR "behaviours"[All Fields] OR "behaviors"[All Fields] OR "pattern"[All Fields] OR "pattern s"[All Fields] OR "patternability"[All Fields] OR "patternable"[All Fields] OR "patterned"[All Fields] OR "patterning"[All Fields] OR "patternings"[All Fields] OR "patterns"[All Fields]) AND ("locomotor"[All Fields] OR "locomotors"[All Fields])) OR (("locomote"[All Fields] OR "locomoted"[All Fields] OR "locomoter"[All Fields] OR "locomotes"[All Fields] OR "locomoting"[All Fields] OR "locomotion"[MeSH Terms] OR "locomotion"[All Fields] OR "locomotions"[All Fields] OR "locomotive"[All Fields] OR "locomotives"[All Fields]) AND ("behavior"[MeSH Terms] OR "behavior"[All Fields] OR "behavioral"[All Fields] OR "behavioural"[All Fields] OR "behavior s"[All Fields] OR "behaviorally"[All Fields] OR "behaviour"[All Fields] OR "behaviourally"[All Fields] OR "behaviours"[All Fields] OR "behaviors"[All Fields] OR "pattern"[All Fields] OR "pattern s"[All Fields] OR "patternability"[All Fields] OR "patternable"[All Fields] OR "patterned"[All Fields] OR "patterning"[All Fields] OR "patternings"[All Fields] OR "patterns"[All Fields])) OR ("locomotion"[MeSH Terms] OR "locomotion"[All Fields] OR ("locomotor"[All Fields] AND "activity"[All Fields]) OR "locomotor activity"[All Fields]) OR (("locomotor"[All Fields] OR "locomotors"[All Fields]) AND ("behavior"[MeSH Terms] OR "behavior"[All Fields] OR "behavioral"[All Fields] OR "behavioural"[All Fields] OR "behavior s"[All Fields] OR "behaviorally"[All Fields] OR "behaviour"[All Fields] OR "behaviourally"[All Fields] OR "behaviours"[All Fields] OR "behaviors"[All Fields] OR "pattern"[All Fields] OR "pattern s"[All Fields] OR "patternability"[All Fields] OR "patternable"[All Fields] OR "patterned"[All Fields] OR "patterning"[All Fields] OR "patternings"[All Fields] OR "patterns"[All Fields])) OR (("locomotor"[All Fields] OR "locomotors"[All Fields]) AND ("behavior"[MeSH Terms] OR "behavior"[All Fields] OR "behavioral"[All Fields] OR "behavioural"[All Fields] OR "behavior s"[All Fields] OR "behaviorally"[All Fields] OR "behaviour"[All Fields] OR "behaviourally"[All Fields] OR "behaviours"[All Fields] OR "behaviors"[All Fields] OR "pattern"[All Fields] OR "pattern s"[All Fields] OR "patternability"[All Fields] OR "patternable"[All Fields] OR "patterned"[All Fields] OR "patterning"[All Fields] OR "patternings"[All Fields] OR "patterns"[All Fields])) OR (("locomotor"[All Fields] OR "locomotors"[All Fields]) AND ("response"[All Fields] OR "responses"[All Fields] OR "responsive"[All Fields] OR "responsiveness"[All Fields] OR "responsivenesses"[All Fields] OR "responsives"[All Fields] OR "responsivities"[All Fields] OR "responsivity"[All Fields])) OR ("motor activity"[MeSH Terms] OR ("motor"[All Fields] AND "activity"[All Fields]) OR "motor activity"[All Fields] OR ("motor"[All Fields] AND "behavior"[All Fields]) OR "motor behavior"[All Fields]) OR ("motor activity"[MeSH Terms] OR ("motor"[All Fields] AND "activity"[All Fields]) OR "motor activity"[All Fields] OR ("motor"[All Fields] AND "behaviour"[All Fields]) OR "motor behaviour"[All Fields]) OR ("walked"[All Fields] OR "walking"[MeSH Terms] OR "walking"[All Fields] OR "walks"[All Fields]) OR ("running"[MeSH Terms] OR "running"[All Fields] OR "runnings"[All Fields]) OR ("mobilities"[All Fields] OR "range

of motion, articular"[MeSH Terms] OR ("range"[All Fields] AND "motion"[All Fields] AND "articular"[All Fields]) OR "articular range of motion"[All Fields] OR "mobility"[All Fields]) OR ("ambulant"[All Fields] OR "ambulate"[All Fields] OR "ambulated"[All Fields] OR "ambulates"[All Fields] OR "ambulating"[All Fields] OR "ambulations"[All Fields] OR "ambulator"[All Fields] OR "ambulators"[All Fields] OR "walking"[MeSH Terms] OR "walking"[All Fields] OR "ambulation"[All Fields]) OR ("early ambulation"[MeSH Terms] OR ("early"[All Fields] AND "ambulation"[All Fields]) OR "early ambulation"[All Fields]) OR ("early mobilisation"[All Fields] OR "early ambulation"[MeSH Terms] OR ("early"[All Fields] AND "ambulation"[All Fields]) OR "early ambulation"[All Fields] OR ("early"[All Fields] AND "mobilization"[All Fields]) OR "early mobilization"[All Fields]) OR ("early mobilisation"[All Fields] OR "early ambulation"[MeSH Terms] OR ("early"[All Fields] AND "ambulation"[All Fields]) OR "early ambulation"[All Fields] OR ("early"[All Fields] AND "mobilization"[All Fields]) OR "early mobilization"[All Fields]) OR ("mobilisation"[All Fields] OR "mobilisations"[All Fields] OR "mobilise"[All Fields] OR "mobilised"[All Fields] OR "mobiliser"[All Fields] OR "mobilisers"[All Fields] OR "mobilises"[All Fields] OR "mobilising"[All Fields] OR "mobilization"[All Fields] OR "mobilizations"[All Fields] OR "mobilize"[All Fields] OR "mobilized"[All Fields] OR "mobilizer"[All Fields] OR "mobilizers"[All Fields] OR "mobilizes"[All Fields] OR "mobilizing"[All Fields]) OR ("mobilisation"[All Fields] OR "mobilisations"[All Fields] OR "mobilise"[All Fields] OR "mobilised"[All Fields] OR "mobiliser"[All Fields] OR "mobilisers"[All Fields] OR "mobilises"[All Fields] OR "mobilising"[All Fields] OR "mobilization"[All Fields] OR "mobilizations"[All Fields] OR "mobilize"[All Fields] OR "mobilized"[All Fields] OR "mobilizer"[All Fields] OR "mobilizers"[All Fields] OR "mobilizes"[All Fields] OR "mobilizing"[All Fields]) OR ("posture"[MeSH Terms] OR "posture"[All Fields] OR ("body"[All Fields] AND "position"[All Fields]) OR "body position"[All Fields]) OR (("postural"[All Fields] OR "posturally"[All Fields] OR "posture"[MeSH Terms] OR "posture"[All Fields] OR "postures"[All Fields] OR "postured"[All Fields] OR "posturing"[All Fields]) AND ("transfer"[All Fields] OR "transferability"[All Fields] OR "transferable"[All Fields] OR "transfered"[All Fields] OR "transfering"[All Fields] OR "transferred"[All Fields] OR "transferring"[All Fields] OR "transfers"[All Fields])) OR (("sitting position"[MeSH Terms] OR ("sitting"[All Fields] AND "position"[All Fields]) OR "sitting position"[All Fields] OR "sit"[All Fields]) AND ("stand"[All Fields] OR "standing position"[MeSH Terms] OR ("standing"[All Fields] AND "position"[All Fields]) OR "standing position"[All Fields] OR "standing"[All Fields] OR "standings"[All Fields] OR "stands"[All Fields])) OR ("stand"[All Fields] OR "standing position"[MeSH Terms] OR ("standing"[All Fields] AND "position"[All Fields]) OR "standing position"[All Fields] OR "standing"[All Fields] OR "standings"[All Fields] OR "stands"[All Fields]) OR "orthoposition"[All Fields] OR ("dizziness"[MeSH Terms] OR "dizziness"[All Fields] OR "orthostasis"[All Fields]) OR (("orthostatically"[All Fields] OR "orthostatics"[All Fields] OR "orthostatism"[All Fields] OR "standing position"[MeSH Terms] OR ("standing"[All Fields] AND "position"[All Fields]) OR "standing position"[All Fields] OR "orthostatic"[All Fields]) AND ("patient positioning"[MeSH Terms] OR ("patient"[All Fields] AND "positioning"[All Fields]) OR "patient positioning"[All Fields] OR "positioning"[All Fields] OR "position"[All Fields] OR "position s"[All Fields] OR "positional"[All Fields] OR "positioned"[All Fields] OR "positionings"[All Fields] OR

"positions"[All Fields])) OR ("orthostatically"[All Fields] OR "orthostatics"[All Fields] OR "orthostatism"[All Fields] OR "standing position"[MeSH Terms] OR ("standing"[All Fields] AND "position"[All Fields]) OR "standing position"[All Fields] OR "orthostatic"[All Fields]) OR ("orthostatically"[All Fields] OR "orthostatics"[All Fields] OR "orthostatism"[All Fields] OR "standing position"[MeSH Terms] OR ("standing"[All Fields] AND "position"[All Fields]) OR "standing position"[All Fields] OR "orthostatic"[All Fields]) OR ("stance"[All Fields] OR "stances"[All Fields]) OR ("standing position"[MeSH Terms] OR ("standing"[All Fields] AND "position"[All Fields]) OR "standing position"[All Fields]) OR (("upright"[All Fields] OR "uprightness"[All Fields] OR "uprights"[All Fields]) AND ("patient positioning"[MeSH Terms] OR ("patient"[All Fields] AND "positioning"[All Fields]) OR "patient positioning"[All Fields] OR "positioning"[All Fields] OR "position"[All Fields] OR "position s"[All Fields] OR "positional"[All Fields] OR "positioned"[All Fields] OR "positionings"[All Fields] OR "positions"[All Fields]))) AND ((((((("functional"[All Fields] OR "functional s"[All Fields] OR "functionalities"[All Fields] OR "functionality"[All Fields] OR "functionalization"[All Fields] OR "functionalizations"[All Fields] OR "functionalize"[All Fields] OR "functionalized"[All Fields] OR "functionalizes"[All Fields] OR "functionalizing"[All Fields] OR "functionally"[All Fields] OR "functionals"[All Fields] OR "functioned"[All Fields] OR "functioning"[All Fields] OR "functionings"[All Fields] OR "functions"[All Fields] OR "physiology"[MeSH Subheading] OR "physiology"[All Fields] OR "function"[All Fields] OR "physiology"[MeSH Terms]) AND ("spectroscopy, near infrared"[MeSH Terms] OR ("spectroscopy"[All Fields] AND "near-infrared"[All Fields]) OR "near-infrared spectroscopy"[All Fields] OR ("near"[All Fields] AND "infrared"[All Fields] AND "spectroscopy"[All Fields]) OR "near infrared spectroscopy"[All Fields])) OR ("fnir"[All Fields] AND ("spectroscopies"[All Fields] OR "spectroscopy s"[All Fields] OR "spectrum analysis"[MeSH Terms] OR ("spectrum"[All Fields] AND "analysis"[All Fields]) OR "spectrum analysis"[All Fields] OR "spectroscopy"[All Fields])) OR "fnirs"[All Fields] OR (("functional"[All Fields] OR "functional s"[All Fields] OR "functionalities"[All Fields] OR "functionality"[All Fields] OR "functionalization"[All Fields] OR "functionalizations"[All Fields] OR "functionalize"[All Fields] OR "functionalized"[All Fields] OR "functionalizes"[All Fields] OR "functionalizing"[All Fields] OR "functionally"[All Fields] OR "functionals"[All Fields] OR "functioned"[All Fields] OR "functioning"[All Fields] OR "functionings"[All Fields] OR "functions"[All Fields] OR "physiology"[MeSH Subheading] OR "physiology"[All Fields] OR "function"[All Fields] OR "physiology"[MeSH Terms]) AND ("spectroscopy, near infrared"[MeSH Terms] OR ("spectroscopy"[All Fields] AND "near-infrared"[All Fields]) OR "near-infrared spectroscopy"[All Fields] OR ("nir"[All Fields] AND "spectroscopy"[All Fields]) OR "nir spectroscopy"[All Fields])) OR (("functional"[All Fields] OR "functional s"[All Fields] OR "functionalities"[All Fields] OR "functionality"[All Fields] OR "functionalization"[All Fields] OR "functionalizations"[All Fields] OR "functionalize"[All Fields] OR "functionalized"[All Fields] OR "functionalizes"[All Fields] OR "functionalizing"[All Fields] OR "functionally"[All Fields] OR "functionals"[All Fields] OR "functioned"[All Fields] OR "functioning"[All Fields] OR "functionings"[All Fields] OR "functions"[All Fields] OR "physiology"[MeSH Subheading] OR "physiology"[All Fields] OR "function"[All Fields] OR "physiology"[MeSH Terms]) AND "near"[All Fields] AND "infra-red"[All

Fields] AND ("spectroscopies"[All Fields] OR "spectroscopy s"[All Fields] OR "spectrum analysis"[MeSH Terms] OR ("spectrum"[All Fields] AND "analysis"[All Fields]) OR "spectrum analysis"[All Fields] OR "spectroscopy"[All Fields])) OR (("functional"[All Fields] OR "functional s"[All Fields] OR "functionalities"[All Fields] OR "functionality"[All Fields] OR "functionalization"[All Fields] OR "functionalizations"[All Fields] OR "functionalize"[All Fields] OR "functionalized"[All Fields] OR "functionalizes"[All Fields] OR "functionalizing"[All Fields] OR "functionally"[All Fields] OR "functionals"[All Fields] OR "functioned"[All Fields] OR "functioning"[All Fields] OR "functionings"[All Fields] OR "functions"[All Fields] OR "physiology"[MeSH Subheading] OR "physiology"[All Fields] OR "function"[All Fields] OR "physiology"[MeSH Terms]) AND "near-IR"[All Fields] AND ("spectroscopies"[All Fields] OR "spectroscopy s"[All Fields] OR "spectrum analysis"[MeSH Terms] OR ("spectrum"[All Fields] AND "analysis"[All Fields]) OR "spectrum analysis"[All Fields] OR "spectroscopy"[All Fields])) OR (("functional"[All Fields] OR "functional s"[All Fields] OR "functionalities"[All Fields] OR "functionality"[All Fields] OR "functionalization"[All Fields] OR "functionalizations"[All Fields] OR "functionalize"[All Fields] OR "functionalized"[All Fields] OR "functionalizes"[All Fields] OR "functionalizing"[All Fields] OR "functionally"[All Fields] OR "functionals"[All Fields] OR "functioned"[All Fields] OR "functioning"[All Fields] OR "functionings"[All Fields] OR "functions"[All Fields] OR "physiology"[MeSH Subheading] OR "physiology"[All Fields] OR "function"[All Fields] OR "physiology"[MeSH Terms]) AND ("spectroscopy, near infrared"[MeSH Terms] OR ("spectroscopy"[All Fields] AND "near-infrared"[All Fields]) OR "near-infrared spectroscopy"[All Fields] OR ("near"[All Fields] AND "infrared"[All Fields] AND "spectrometry"[All Fields]) OR "near infrared spectrometry"[All Fields])) OR (("functional"[All Fields] OR "functional s"[All Fields] OR "functionalities"[All Fields] OR "functionality"[All Fields] OR "functionalization"[All Fields] OR "functionalizations"[All Fields] OR "functionalize"[All Fields] OR "functionalized"[All Fields] OR "functionalizes"[All Fields] OR "functionalizing"[All Fields] OR "functionally"[All Fields] OR "functionals"[All Fields] OR "functioned"[All Fields] OR "functioning"[All Fields] OR "functionings"[All Fields] OR "functions"[All Fields] OR "physiology"[MeSH Subheading] OR "physiology"[All Fields] OR "function"[All Fields] OR "physiology"[MeSH Terms]) AND "near-infrared"[All Fields] AND ("spectrophotometries"[All Fields] OR "spectrophotometry"[MeSH Terms] OR "spectrophotometry"[All Fields])) OR (("functional"[All Fields] OR "functional s"[All Fields] OR "functionalities"[All Fields] OR "functionality"[All Fields] OR "functionalization"[All Fields] OR "functionalizations"[All Fields] OR "functionalize"[All Fields] OR "functionalized"[All Fields] OR "functionalizes"[All Fields] OR "functionalizing"[All Fields] OR "functionally"[All Fields] OR "functionals"[All Fields] OR "functioned"[All Fields] OR "functioning"[All Fields] OR "functionings"[All Fields] OR "functions"[All Fields] OR "physiology"[MeSH Subheading] OR "physiology"[All Fields] OR "function"[All Fields] OR "physiology"[MeSH Terms]) AND "near-infrared"[All Fields] AND ("spectroscope"[All Fields] OR "spectroscopes"[All Fields] OR "spectroscopic"[All Fields] OR "spectroscopical"[All Fields] OR "spectroscopically"[All Fields]) AND ("investigated"[All Fields] OR "investigates"[All Fields] OR "investigating"[All Fields] OR "investigation"[All Fields] OR "investigations"[All Fields] OR "investigative"[All

Fields] OR "investigator s"[All Fields] OR "research personnel"[MeSH Terms] OR ("research"[All Fields] AND "personnel"[All Fields]) OR "research personnel"[All Fields] OR "investigator"[All Fields] OR "investigators"[All Fields])) OR (("functional"[All Fields] OR "functional s"[All Fields] OR "functionalities"[All Fields] OR "functionality"[All Fields] OR "functionalization"[All Fields] OR "functionalizations"[All Fields] OR "functionalize"[All Fields] OR "functionalized"[All Fields] OR "functionalizes"[All Fields] OR "functionalizing"[All Fields] OR "functionally"[All Fields] OR "functionals"[All Fields] OR "functioned"[All Fields] OR "functioning"[All Fields] OR "functionings"[All Fields] OR "functions"[All Fields] OR "physiology"[MeSH Subheading] OR "physiology"[All Fields] OR "function"[All Fields] OR "physiology"[MeSH Terms]) AND "near-infrared"[All Fields] AND ("spectroscope"[All Fields] OR "spectroscopes"[All Fields] OR "spectroscopic"[All Fields] OR "spectroscopical"[All Fields] OR "spectroscopically"[All Fields]) AND ("studies"[All Fields] OR "study"[All Fields] OR "study s"[All Fields] OR "studying"[All Fields] OR "studys"[All Fields])) OR (("functional"[All Fields] OR "functional s"[All Fields] OR "functionalities"[All Fields] OR "functionality"[All Fields] OR "functionalization"[All Fields] OR "functionalizations"[All Fields] OR "functionalize"[All Fields] OR "functionalized"[All Fields] OR "functionalizes"[All Fields] OR "functionalizing"[All Fields] OR "functionally"[All Fields] OR "functionals"[All Fields] OR "functioned"[All Fields] OR "functioning"[All Fields] OR "functionings"[All Fields] OR "functions"[All Fields] OR "physiology"[MeSH Subheading] OR "physiology"[All Fields] OR "function"[All Fields] OR "physiology"[MeSH Terms]) AND ("spectroscopy, near infrared"[MeSH Terms] OR ("spectroscopy"[All Fields] AND "near-infrared"[All Fields]) OR "near-infrared spectroscopy"[All Fields] OR ("near"[All Fields] AND "infrared"[All Fields] AND "spectroscopy"[All Fields]) OR "near infrared spectroscopy"[All Fields])) OR (("functional"[All Fields] OR "functional s"[All Fields] OR "functionalities"[All Fields] OR "functionality"[All Fields] OR "functionalization"[All Fields] OR "functionalizations"[All Fields] OR "functionalize"[All Fields] OR "functionalized"[All Fields] OR "functionalizes"[All Fields] OR "functionalizing"[All Fields] OR "functionally"[All Fields] OR "functionals"[All Fields] OR "functioned"[All Fields] OR "functioning"[All Fields] OR "functionings"[All Fields] OR "functions"[All Fields] OR "physiology"[MeSH Subheading] OR "physiology"[All Fields] OR "function"[All Fields] OR "physiology"[MeSH Terms]) AND ("spectroscopy, near infrared"[MeSH Terms] OR ("spectroscopy"[All Fields] AND "near-infrared"[All Fields]) OR "near-infrared spectroscopy"[All Fields] OR ("near"[All Fields] AND "infrared"[All Fields] AND "spectroscopy"[All Fields]) OR "near infrared spectroscopy"[All Fields])) OR ("electroencephalography"[MeSH Terms] OR "electroencephalography"[All Fields] OR "electroencephalogram"[All Fields] OR "electroencephalograms"[All Fields]) OR ("electroencephalography"[MeSH Terms] OR "electroencephalography"[All Fields] OR "eeg"[All Fields]) OR (("brain"[MeSH Terms] OR "brain"[All Fields] OR "brains"[All Fields] OR "brain s"[All Fields]) AND ("activable"[All Fields] OR "activate"[All Fields] OR "activated"[All Fields] OR "activates"[All Fields] OR "activating"[All Fields] OR "activation"[All Fields] OR "activations"[All Fields] OR "activator"[All Fields] OR "activator s"[All Fields] OR "activators"[All Fields] OR "active"[All Fields] OR "actived"[All Fields] OR "actively"[All Fields] OR "actives"[All Fields] OR "activities"[All Fields] OR "activity s"[All Fields] OR "activitys"[All Fields] OR "motor

activity"[MeSH Terms] OR ("motor"[All Fields] AND "activity"[All Fields]) OR "motor activity"[All Fields] OR "activity"[All Fields])) OR (("brain"[MeSH Terms] OR "brain"[All Fields] OR "brains"[All Fields] OR "brain s"[All Fields]) AND ("electricity"[MeSH Terms] OR "electricity"[All Fields] OR "electric"[All Fields] OR "electrical"[All Fields] OR "electrically"[All Fields] OR "electrics"[All Fields]) AND ("activable"[All Fields] OR "activate"[All Fields] OR "activated"[All Fields] OR "activates"[All Fields] OR "activating"[All Fields] OR "activation"[All Fields] OR "activations"[All Fields] OR "activator"[All Fields] OR "activator s"[All Fields] OR "activators"[All Fields] OR "active"[All Fields] OR "actived"[All Fields] OR "actively"[All Fields] OR "actives"[All Fields] OR "activities"[All Fields] OR "activity s"[All Fields] OR "activitys"[All Fields] OR "motor activity"[MeSH Terms] OR ("motor"[All Fields] AND "activity"[All Fields]) OR "motor activity"[All Fields] OR "activity"[All Fields])) OR (("brain"[MeSH Terms] OR "brain"[All Fields] OR "brains"[All Fields] OR "brain s"[All Fields]) AND ("electricity"[MeSH Terms] OR "electricity"[All Fields] OR "electric"[All Fields] OR "electrical"[All Fields] OR "electrically"[All Fields] OR "electrics"[All Fields]) AND ("activable"[All Fields] OR "activate"[All Fields] OR "activated"[All Fields] OR "activates"[All Fields] OR "activating"[All Fields] OR "activation"[All Fields] OR "activations"[All Fields] OR "activator"[All Fields] OR "activator s"[All Fields] OR "activators"[All Fields] OR "active"[All Fields] OR "actived"[All Fields] OR "actively"[All Fields] OR "actives"[All Fields] OR "activities"[All Fields] OR "activity s"[All Fields] OR "activitys"[All Fields] OR "motor activity"[MeSH Terms] OR ("motor"[All Fields] AND "activity"[All Fields]) OR "motor activity"[All Fields] OR "activity"[All Fields])) OR ("brain waves"[MeSH Terms] OR ("brain"[All Fields] AND "waves"[All Fields]) OR "brain waves"[All Fields] OR ("brain"[All Fields] AND "wave"[All Fields]) OR "brain wave"[All Fields]) OR ("brain waves"[MeSH Terms] OR ("brain"[All Fields] AND "waves"[All Fields]) OR "brain waves"[All Fields]) OR ("brain waves"[MeSH Terms] OR ("brain"[All Fields] AND "waves"[All Fields]) OR "brain waves"[All Fields] OR "brainwave"[All Fields] OR "brainwaves"[All Fields]) OR ("brain waves"[MeSH Terms] OR ("brain"[All Fields] AND "waves"[All Fields]) OR "brain waves"[All Fields] OR "brainwave"[All Fields] OR "brainwaves"[All Fields]) OR "e e g"[All Fields] OR (("electroencephalography"[MeSH Terms] OR "electroencephalography"[All Fields] OR "eeg"[All Fields]) AND ("activable"[All Fields] OR "activate"[All Fields] OR "activated"[All Fields] OR "activates"[All Fields] OR "activating"[All Fields] OR "activation"[All Fields] OR "activations"[All Fields] OR "activator"[All Fields] OR "activator s"[All Fields] OR "activators"[All Fields] OR "active"[All Fields] OR "actived"[All Fields] OR "actively"[All Fields] OR "actives"[All Fields] OR "activities"[All Fields] OR "activity s"[All Fields] OR "activitys"[All Fields] OR "motor activity"[MeSH Terms] OR ("motor"[All Fields] AND "activity"[All Fields]) OR "motor activity"[All Fields] OR "activity"[All Fields])) OR (("electroencephalography"[MeSH Terms] OR "electroencephalography"[All Fields] OR "eeg"[All Fields]) AND ("analysis"[MeSH Subheading] OR "analysis"[All Fields])) OR (("electricity"[MeSH Terms] OR "electricity"[All Fields] OR "electric"[All Fields] OR "electrical"[All Fields] OR "electrically"[All Fields] OR "electrics"[All Fields]) AND ("encephalogram"[All Fields] OR "encephalograms"[All Fields])) OR (("electricity"[MeSH Terms] OR "electricity"[All Fields] OR "electric"[All Fields] OR "electrical"[All Fields] OR "electrically"[All Fields] OR "electrics"[All Fields]) AND ("encephalogram"[All Fields]

OR "encephalograms"[All Fields])) OR ("electro"[All Fields] AND ("encephalogram"[All Fields] OR "encephalograms"[All Fields])) OR ("electroencephalography"[MeSH Terms] OR "electroencephalography"[All Fields] OR "electroencephalogram"[All Fields] OR "electroencephalograms"[All Fields]) OR (("isoelectric"[All Fields] OR "isoelectrical"[All Fields] OR "isoelectrically"[All Fields]) AND ("electroencephalography"[MeSH Terms] OR "electroencephalography"[All Fields] OR "eeg"[All Fields])) OR ("diffusion tensor imaging"[MeSH Terms] OR ("diffusion"[All Fields] AND "tensor"[All Fields] AND "imaging"[All Fields]) OR "diffusion tensor imaging"[All Fields]) OR "dti"[All Fields]) AND ("diffusion tensor imaging"[MeSH Terms] OR ("diffusion"[All Fields] AND "tensor"[All Fields] AND "imaging"[All Fields]) OR "diffusion tensor imaging"[All Fields])) OR "dtt"[All Fields]) AND (("diffusable"[All Fields] OR "diffusant"[All Fields] OR "diffusants"[All Fields] OR "diffuse"[All Fields] OR "diffusely"[All Fields] OR "diffuses"[All Fields] OR "diffusibility"[All Fields] OR "diffusible"[All Fields] OR "diffusion"[MeSH Terms] OR "diffusion"[All Fields] OR "diffused"[All Fields] OR "diffusing"[All Fields] OR "diffusions"[All Fields] OR "diffusive"[All Fields] OR "diffusively"[All Fields] OR "diffusivities"[All Fields] OR "diffusivity"[All Fields]) AND ("tensor"[All Fields] OR "tensor s"[All Fields] OR "tensors"[All Fields]) AND ("tractographies"[All Fields] OR "tractography"[All Fields]))) OR ("diffusion tensor imaging"[MeSH Terms] OR ("diffusion"[All Fields] AND "tensor"[All Fields] AND "imaging"[All Fields]) OR "diffusion tensor imaging"[All Fields] OR ("diffusion"[All Fields] AND "tensor"[All Fields] AND "mri"[All Fields]) OR "diffusion tensor mri"[All Fields]) OR ("diffusion tensor imaging"[MeSH Terms] OR ("diffusion"[All Fields] AND "tensor"[All Fields] AND "imaging"[All Fields]) OR "diffusion tensor imaging"[All Fields]) OR ("diffusion tensor imaging"[MeSH Terms] OR ("diffusion"[All Fields] AND "tensor"[All Fields] AND "imaging"[All Fields]) OR "diffusion tensor imaging"[All Fields] OR ("diffusion"[All Fields] AND "tensor"[All Fields] AND "magnetic"[All Fields] AND "resonance"[All Fields] AND "imaging"[All Fields]) OR "diffusion tensor magnetic resonance imaging"[All Fields]) OR (("diffusable"[All Fields] OR "diffusant"[All Fields] OR "diffusants"[All Fields] OR "diffuse"[All Fields] OR "diffusely"[All Fields] OR "diffuses"[All Fields] OR "diffusibility"[All Fields] OR "diffusible"[All Fields] OR "diffusion"[MeSH Terms] OR "diffusion"[All Fields] OR "diffused"[All Fields] OR "diffusing"[All Fields] OR "diffusions"[All Fields] OR "diffusive"[All Fields] OR "diffusively"[All Fields] OR "diffusivities"[All Fields] OR "diffusivity"[All Fields]) AND ("tensor"[All Fields] OR "tensor s"[All Fields] OR "tensors"[All Fields]) AND ("tractographies"[All Fields] OR "tractography"[All Fields])) OR (("magnetic resonance spectroscopy"[MeSH Terms] OR ("magnetic"[All Fields] AND "resonance"[All Fields] AND "spectroscopy"[All Fields]) OR "magnetic resonance spectroscopy"[All Fields] OR ("magnetic"[All Fields] AND "resonance"[All Fields]) OR "magnetic resonance"[All Fields]) AND ("diffusion tensor imaging"[MeSH Terms] OR ("diffusion"[All Fields] AND "tensor"[All Fields] AND "imaging"[All Fields]) OR "diffusion tensor imaging"[All Fields])) OR ("magnetic resonance imaging"[MeSH Terms] OR ("magnetic"[All Fields] AND "resonance"[All Fields] AND "imaging"[All Fields]) OR "magnetic resonance imaging"[All Fields] OR ("functional"[All Fields] AND "magnetic"[All Fields] AND "resonance"[All Fields] AND "imaging"[All Fields]) OR "functional magnetic resonance imaging"[All Fields]) OR "r fmri"[All Fields] OR ("magnetic resonance imaging"[MeSH Terms] OR ("magnetic"[All Fields] AND

"resonance"[All Fields] AND "imaging"[All Fields]) OR "magnetic resonance imaging"[All Fields] OR "fmri"[All Fields]) OR ("magnetic resonance imaging"[MeSH Terms] OR ("magnetic"[All Fields] AND "resonance"[All Fields] AND "imaging"[All Fields]) OR "magnetic resonance imaging"[All Fields] OR ("functional"[All Fields] AND "mri"[All Fields]) OR "functional mri"[All Fields]) OR ("magnetic resonance imaging"[MeSH Terms] OR ("magnetic"[All Fields] AND "resonance"[All Fields] AND "imaging"[All Fields]) OR "magnetic resonance imaging"[All Fields] OR ("functional"[All Fields] AND "magnetic"[All Fields] AND "resonance"[All Fields] AND "imaging"[All Fields]) OR "functional magnetic resonance imaging"[All Fields]) OR ("magnetic resonance imaging"[MeSH Terms] OR ("magnetic"[All Fields] AND "resonance"[All Fields] AND "imaging"[All Fields]) OR "magnetic resonance imaging"[All Fields] OR ("magnetic"[All Fields] AND "resonance"[All Fields] AND "imaging"[All Fields] AND "functional"[All Fields]) OR "magnetic resonance imaging functional"[All Fields]) OR (("rest"[MeSH Terms] OR "rest"[All Fields] OR "rested"[All Fields] OR "resting"[All Fields]) AND ("state"[All Fields] OR "state s"[All Fields] OR "stated"[All Fields] OR "states"[All Fields] OR "stating"[All Fields]) AND ("magnetic resonance imaging"[MeSH Terms] OR ("magnetic"[All Fields] AND "resonance"[All Fields] AND "imaging"[All Fields]) OR "magnetic resonance imaging"[All Fields] OR ("functional"[All Fields] AND "magnetic"[All Fields] AND "resonance"[All Fields] AND "imaging"[All Fields]) OR "functional magnetic resonance imaging"[All Fields])) OR "rsfmri"[All Fields] OR ("neuroimage"[All Fields] OR "neuroimaged"[All Fields] OR "neuroimagers"[All Fields] OR "neuroimages"[All Fields] OR "neuroimaging"[MeSH Terms] OR "neuroimaging"[All Fields] OR "neuroimagings"[All Fields]) OR (("neural"[All Fields] OR "neuralization"[All Fields] OR "neuralize"[All Fields] OR "neuralized"[All Fields] OR "neuralizes"[All Fields] OR "neuralizing"[All Fields] OR "neurally"[All Fields]) AND ("image"[All Fields] OR "image s"[All Fields] OR "imaged"[All Fields] OR "imager"[All Fields] OR "imager s"[All Fields] OR "imagers"[All Fields] OR "images"[All Fields] OR "imaging"[All Fields] OR "imaging s"[All Fields] OR "imagings"[All Fields])) OR "neuro imaging"[All Fields] OR ("neuroimage"[All Fields] OR "neuroimaged"[All Fields] OR "neuroimagers"[All Fields] OR "neuroimages"[All Fields] OR "neuroimaging"[MeSH Terms] OR "neuroimaging"[All Fields] OR "neuroimagings"[All Fields]) OR ("magnetoencephalography"[MeSH Terms] OR "magnetoencephalography"[All Fields]) OR ("transcranial magnetic stimulation"[MeSH Terms] OR ("transcranial"[All Fields] AND "magnetic"[All Fields] AND "stimulation"[All Fields]) OR "transcranial magnetic stimulation"[All Fields]) OR ("transcranial magnetic stimulation"[MeSH Terms] OR ("transcranial"[All Fields] AND "magnetic"[All Fields] AND "stimulation"[All Fields]) OR "transcranial magnetic stimulation"[All Fields] OR ("magnetic"[All Fields] AND "stimulation"[All Fields] AND "transcranial"[All Fields]) OR "magnetic stimulation transcranial"[All Fields]) OR ("transcranial magnetic stimulation"[MeSH Terms] OR ("transcranial"[All Fields] AND "magnetic"[All Fields] AND "stimulation"[All Fields]) OR "transcranial magnetic stimulation"[All Fields] OR ("stimulation"[All Fields] AND "transcranial"[All Fields] AND "magnetic"[All Fields]) OR "stimulation transcranial magnetic"[All Fields]) OR ("transcranial magnetic stimulation"[MeSH Terms] OR ("transcranial"[All Fields] AND "magnetic"[All Fields] AND "stimulation"[All Fields]) OR "transcranial magnetic stimulation"[All Fields]) OR (("cerebrally"[All Fields] OR "cerebrum"[MeSH Terms] OR "cerebrum"[All Fields] OR "cerebral"[All Fields] OR

"brain"[MeSH Terms] OR "brain"[All Fields]) AND ("activable"[All Fields] OR "activate"[All Fields] OR "activated"[All Fields] OR "activates"[All Fields] OR "activating"[All Fields] OR "activation"[All Fields] OR "activations"[All Fields] OR "activator"[All Fields] OR "activator s"[All Fields] OR "activators"[All Fields] OR "active"[All Fields] OR "actived"[All Fields] OR "actively"[All Fields] OR "actives"[All Fields] OR "activities"[All Fields] OR "activity s"[All Fields] OR "activitys"[All Fields] OR "motor activity"[MeSH Terms] OR ("motor"[All Fields] AND "activity"[All Fields]) OR "motor activity"[All Fields] OR "activity"[All Fields])))) NOT "systematic review"[Title]

EMBASE:

((((('cerebral palsy'/exp OR 'brain palsy' OR 'brain paralysis' OR 'central palsy' OR 'central paralysis' OR 'cerebral palsy' OR 'cerebral paralysis' OR 'cerebral paresis' OR 'diplegia spastica' OR 'encephalopathia infantilis' OR 'palsy, cerebral' OR 'spastic diplegia') AND ('child' OR 'children' OR 'toddler' OR 'toddlers' OR 'adolescent' OR 'teenager' OR ‘adolescents’ OR ‘infants’)) NOT ('adult' OR 'adults' OR 'grown-ups' OR 'grownup' OR 'grownups' OR 'aged' OR 'aged patient' OR 'aged people' OR 'aged person' OR 'aged subject' OR 'elderly' OR 'elderly patient' OR 'elderly people' OR 'elderly person' OR 'elderly subject' OR 'senior citizen' OR 'senium' OR 'older adults' OR 'older people' OR ‘newborn’ OR 'animals, newborn' OR 'human neonate' OR 'human newborn' OR 'neonatal animal' OR 'neonate' OR 'neonate animal' OR 'neonatus' OR 'newborn' OR 'newborn animal' OR 'newborn animals' OR 'newborn baby' OR 'newborn child' OR 'newborn infant' OR 'newly born animal' OR 'newly born baby' OR 'newly born child' OR 'newly born infant' OR ‘fetus’ OR 'fetal period' OR 'fetal phase' OR 'fetal stage' OR 'fetus' OR 'foetal period' OR 'foetal phase' OR 'foetal stage' OR 'foetus') AND ('biped gait' OR 'gait' OR 'gait analysis' OR 'gait training' OR 'pattern, walking' OR 'walking pattern' OR ‘locomotion’ OR 'behavior, locomotor' OR 'behaviour, locomotor' OR 'locomotion pattern' OR 'locomotor activity' OR 'locomotor behavior' OR 'locomotor behaviour' OR 'locomotor response' OR 'motor behavior' OR 'motor behaviour' OR 'walking' OR 'running' OR ‘mobility’ OR 'ambulation' OR 'early ambulation' OR 'early mobilisation' OR 'early mobilization' OR 'mobilisation' OR 'mobilization' OR 'body position' OR 'posture transfer' OR 'sit to stand' OR ‘standing’ OR 'orthoposition' OR 'orthostasis' OR 'orthostatic position' OR 'orthostatics' OR 'orthostatism' OR 'stance' OR 'standing position' OR 'upright position')) AND ('functional near-infrared spectroscopy' OR 'fNIR spectroscopy' OR 'fNIRS' OR 'functional NIR spectroscopy' OR 'functional near infra-red spectroscopy' OR 'functional near-IR spectroscopy' OR 'functional near-infrared spectrometry' OR 'functional near-infrared spectrophotometry' OR 'functional near-infrared spectroscopic investigation' OR 'functional near-infrared spectroscopic study' OR 'functional near-infrared spectroscopy' OR 'functionality near infrared spectroscopy' OR ‘electroencephalogram’ OR 'EEG' OR 'brain activity' OR 'brain electric activity' OR 'brain electrical activity' OR 'brain wave' OR 'brain waves' OR 'brainwave' OR 'brainwaves' OR 'e.e.g.' OR 'eeg activity' OR 'eeg analysis' OR 'electric encephalogram' OR 'electrical encephalogram' OR 'electro encephalogram' OR 'electroencephalogram'

OR 'isoelectric eeg' OR 'diffusion tensor imaging' OR 'DTI (diffusion tensor imaging)' OR 'DTT (diffusion tensor tractography)' OR 'diffusion tensor MRI' OR 'diffusion tensor imaging' OR 'diffusion tensor magnetic resonance imaging' OR 'diffusion tensor tractography' OR 'magnetic resonance diffusion tensor imaging' OR 'functional magnetic resonance imaging' OR 'R-fMRI' OR 'fMRI' OR 'functional MRI' OR 'functional magnetic resonance imaging' OR 'magnetic resonance imaging, functional' OR 'resting state functional magnetic resonance imaging' OR 'rsfMRI' OR ‘neuroimaging’ OR 'neural imaging' OR 'neuro-imaging' OR 'neuroimaging' OR ‘magnetoencephalography’ OR 'transcranial magnetic stimulation' OR 'magnetic stimulation, transcranial' OR 'stimulation, transcranial magnetic' OR 'transcranial magnetic stimulation' OR 'cerebral activity')))
